# Paramedic Management of Non-Traumatic Back Pain in a Large Australian Ambulance Service: A Retrospective Study

**DOI:** 10.1017/S1049023X25000251

**Published:** 2025-04

**Authors:** Simon P. Vella, Chathurani Sigera, Jason C. Bendall, Paul Simpson, Christina Abdel-Shaheed, Michael S. Swain, Chris G. Maher, Gustavo C. Machado

**Affiliations:** 1. Institute for Musculoskeletal Health, Sydney Local Health District, Sydney, New South Wales, Australia; 2. Sydney School of Public Health, Faculty of Medicine and Health, The University of Sydney, Sydney, New South Wales, Australia; 3. Clinical Capability Quality & Safety, New South Wales Ambulance, Sydney, Australia; 4. School of Medicine and Public Health (Anesthesia and Intensive Care), The University of Newcastle, Newcastle, Australia; 5. School of Health Sciences, Western Sydney University, Sydney, New South Wales, Australia; 6. Department of Chiropractic, Faculty of Medicine, Health and Human Sciences, Macquarie University, Sydney, New South Wales, Australia

**Keywords:** ambulance service, back pain, health service, paramedicine, prehospital

## Abstract

**Introduction:**

Non-traumatic back pain commonly leads people to seek health care from paramedics via triple-zero (emergency phone number in Australia), yet the management approaches by providers of ambulance services remain unclear.

**Study Objectives:**

This study aims to investigate paramedic management of non-traumatic back pain in New South Wales (NSW), Australia, including the call characteristics, provisional diagnoses, and the clinical care being delivered by paramedics.

**Methods:**

This study is a retrospective analysis of NSW Ambulance computer-aided dispatch and electronic medical records from January 1, 2017 through December 31, 2022. Adults who sought ambulance service with a chief complaint of back pain, were triaged as non-traumatic back pain, and subsequently received treatment by paramedics were included. Multivariable logistic regression models were used to explore factors associated with primary outcomes; ambulance transport, opioid use, and use of medication combinations were reported as odds ratios (ORs).

**Results:**

There were 73,128 calls to NSW Ambulance with a chief complaint of back pain that were triaged as non-traumatic back pain. Of these, 54,444 (74.4%) were diagnosed with spinal pain, of which 52,825 (97.1%) were categorized by the paramedic as back or neck pain, 1,573 (2.9%) as lumbar radicular pain, and 46 (0.1%) as serious spinal pathology. Eight out of ten patients with spinal pain were transported to emergency departments. The medicine most administered by a paramedic was an opioid (37.4% of patients with spinal pain). Older patients (OR = 1.36; 95% CI, 1.30 to 1.44) were more likely to be transported to an emergency department. Patients with moderate (OR = 4.39; 95% CI, 4.00 to 4.84) and severe pain (OR = 18.90; 95% CI, 17.18 to 20.79) were more likely to be administered an opioid.

**Conclusions:**

Paramedic management of non-traumatic back pain in NSW typically results in the administration of an opioid and transport to an emergency department.

## Introduction

Back pain is the leading cause of *years lived with disability* globally and imposes significant burden to economies and health services.^1,2^ Each year, approximately 660,000 cases of back pain present to emergency departments in the United Kingdom, accounting for 2.8% of all presentations and costing the National Health Service £350 million.^3,4^ Similarly, in Australia, back pain is the sixth most common presentation seen in the emergency department and costs Australian hospitals an estimated AUD$390 million per year, which is often driven by low-value care including lumbar imaging, opioid medication, and unnecessary hospital admission.^5–10^ Most back pain presentations to the emergency department are non-serious (eg, non-specific back pain or lumbar radicular pain), yet one-third of cases arrive via an emergency ambulance. People with back pain who are transported to the emergency department by ambulance are more likely to receive escalation of low-value care including further opioid medicines, laboratory investigations, and hospital admission, which all can have a negative impact on patients through adverse events, like falls and hospital-acquired complications.^5,6^

Ambulance service guidelines for back pain exist, though their recommendations differ to primary care guidelines due to the different settings (ie, emergency health service versus primary care).^11^ Established primary care guidelines for acute back pain recommend self-management, manual therapy, and non-steroidal anti-inflammatory drugs (NSAIDs) or judicious use of weak opioids if NSAIDs are contraindicated.^12^ Ambulance service guidelines for back pain focus on providing paramedics with criteria for emergency department transport (eg, alerting features of serious disease) and alternate referral pathways (eg, medical and allied health services).^11^ Ambulance services that do not have access to specific back pain guidelines use generalized pain management guidelines for paramedics to manage a person’s pain. Back pain and generalized pain management guidelines include pharmacological and non-pharmacological recommendations for care. However, there is little available research that describes paramedic management of back pain in ambulance service and whether guideline recommendations are being followed.

One observational study in England (n = 3,315) evaluated ambulance service use for calls related to back pain. It found that 48% of the calls were for non-spinal conditions (eg, acute abdominal pain, infection), 35% were for spinal pain (eg, non-specific back pain), and 17% of calls required further assessment.^13^ The authors reported that 54% of patients received pharmacological treatment from paramedics, including nitrous-oxide (24.3% of cases), morphine (13.0% of cases), paracetamol (9.0% of cases), and NSAIDs (∼2.4% of cases), and more than two-thirds of patients were transported to the emergency department.^13^ Similarly, an observational study in Spain explored the management of lumbar radicular pain (n = 237) by emergency health services.^14^ The authors reported that 95% of patients received pain medication including muscle relaxants (65% of cases), NSAIDs (54% of cases), opioids (11% of cases), and paracetamol (6%).

There is little available evidence on paramedic management of back pain in Australia. The aims of this study were to investigate paramedic management of non-traumatic back pain in New South Wales (NSW), including call characteristics, the patient profile and their provisional diagnoses, and the interventions provided by paramedics. The study investigated the proportion of cases that were conveyed to an emergency department, administered opioid medication or combination pain medicines, and explored factors associated with these outcomes.

## Methods

### Study Design and Setting

The research team performed a retrospective analysis of ambulance data from NSW from January 2017 through December 2022. Service provider NSW Ambulance (Sydney, NSW, Australia) linked the computer-aided dispatch and electronic medical record datasets and generated de-identified data for analysis. All outcomes presented in this study were available from the linked ambulance dataset. The study was approved by The University of Sydney Human Research Ethics Committee (Sydney, NSW, Australia; Project no. 2023/145) on April 6, 2023.

Currently, NSW has the highest population of any state in Australia (8,834,700 residents as of September 30, 2023), in which NSW Ambulance is the primary ambulance service provider.^15^ At present, NSW Ambulance provides mobile health services and employs 5,765 paramedics across 239 locations in urban, rural, and remote locations with a fleet of 1,241 vehicles.^16^ There are fees associated with ambulance service use, including a call-out charge and a charge per km; however, people on the pension/concession health care and some private health insurance with ambulance coverage can receive ambulance care free-of-charge with no out-of-pocket expense. All emergency services in Australia are accessed by dialing the national emergency number triple-zero. Calls for ambulance in NSW are transferred to one of four control centers. Emergency medical call-takers from NSW Ambulance are accredited with the International Academies of Emergency Dispatch (IAED; Salt Lake City, Utah USA) and use the structured call-taking system Medical Priority Dispatch System (MPDS; supported by IAED).^17^ Triple-zero calls for “back pain” are triaged using the appropriate MPDS protocol. Once triaged, a paramedic team is assigned and dispatched. Paramedics attend the scene and undertake a clinical assessment, make a provisional diagnosis, and initiate management accordingly. Specific protocols from within NSW Ambulance are used to guide paramedic decision making relating to patient care, referral pathways, and discharge destinations (eg, emergency department care).^11^ The generic pain management protocol is used by non-specialist general paramedics and intensive-care paramedics (trained in management of higher-acuity conditions) to manage people presenting to ambulance, according to the severity of their pain. For example, the protocol recommends administration of paracetamol, ibuprofen, and/or methoxyflurane for mild to moderate pain intensity, and morphine, fentanyl, or ketamine for moderate to severe pain. Back pain guidelines exist, but only for specialist extended-care paramedics who have additional training in the management of lower-acuity conditions, like back pain, and have increased capability to treat patients at their home or within the community and have expanded medication scope including oxycodone access. The back pain protocol recommends hot/cold therapy, on-going analgesia (eg, paracetamol, ibuprofen, and/or oxycodone), and referral to primary care.^11^

### Participants and Characteristics

Dispatch and electronic medical records of patients who sought care from NSW Ambulance via triple-zero and were triaged by emergency medical call-takers as having “back pain – non-traumatic” and allocated MPDS determinant 05A01 were analyzed. Participants were included if they were patients of ambulance service aged 16 years and older, capable of providing consent to treatment in accordance with NSW Health (St Leonards, NSW, Australia). Cases were excluded if they: had a non-back-pain-related condition code, were triaged as “back pain-related” complaints but contained irrelevant diagnoses (eg, alcohol intoxication/withdrawal), or were managed using medical or trauma-related protocols, which represents presentations that are due to traumatic injury and/or accident and likely represent different patient characteristics.

The paramedic’s diagnosis was used to categorize cases as spinal pain or non-spinal pain. Spinal pain included back and/or neck pain, lumbar radicular pain, and serious spinal pathology. The number of serious spinal pathology cases were documented but were not included in the analysis due to likely representing different patient characteristics. Non-spinal pain was grouped according to the relevant system: cardiovascular, respiratory, neurological, musculoskeletal, genitourinary, gastrointestinal, psychological, infection, and miscellaneous. Spinal pain cases categorized as back and/or neck pain and lumbar radicular pain were analyzed to describe paramedic management of back pain in this ambulance service.

### Outcomes

Call characteristics, the patient profile, and the management of non-traumatic back pain including pharmacological and non-pharmacological care delivered by paramedics were evaluated. The volume of calls for back pain that were made during business-hours and after-hours, as well as response times, were computed. The proportion of cases and the diagnosis made by paramedics according to patient symptom impression were described. Regarding the management of back pain, the proportion of patients that received non-pharmacological care, including advice and reassurance, education, exercises, patient positioning, and hot/cold therapy, and received pharmacological care were analyzed. Pharmacological care was analyzed according to the Anatomical Therapeutic Chemical (ATC) classification system and included paracetamol, NSAIDs, anesthetics, opioids, and benzodiazepines. The proportion of patients that received each medication type, as well as each type of opioid including morphine, fentanyl, paracetamol-codeine, and oxycodone, were also analyzed, and the proportion of patients with back pain that received combined medicines were described. The five categories of combined medicines used were: (1) simple analgesics (ie, paracetamol and NSAIDs); (2) simple analgesics and anesthetics (ie, paracetamol or NSAIDs and methoxyflurane); (3) simple analgesics and opioids (eg, paracetamol or NSAID and morphine); (4) anesthetics and opioids (eg, methoxyflurane and morphine); and (5) opioids and benzodiazepines (eg, morphine and midazolam).

### Data Collection and Handling

Calls between 9:00am to 5:00pm, Monday through Friday were defined as calls “during working hours.” The patient’s postcode was used to estimate socioeconomic status by geographic area using Socio-Economic Indexes for Areas (SEIFA); SEIFA combines census data such as income, education, employment, occupation, housing, and family structure to determine socio-economic status of an area. The SEIFA categories one to five were defined as low socioeconomic areas and the SEIFA categories six to ten were classed as high socioeconomic areas. The problem “secondary-triage” was used to describe the proportion of patients transferred to the Virtual Call Care Centre (VCCC) for management, a pathway whereby clinicians such as doctors, nurses, and extended-care paramedics manage low-acuity cases virtually, or being managed by a third-party secondary-triage provider. The protocol assigned to a case by a paramedic was used to determine alternate referral pathways including referral to extended-care paramedics or recommendations for the patient to seek further care (eg, general practitioner, medical or allied health services). Baseline pain intensity (on a zero-to-ten verbal numeric rating scale) was categorized as mild (one-to-three), moderate (four-to-seven), and severe (eight-to-ten). Priority codes P1 (immediate response <30 minutes) and P2 (response within 60 minutes) described the urgency of ambulance response. All time variables were reported in minutes and included time from call to ambulance dispatch, time from dispatch to ambulance arrival on scene, duration of care (ie, time from ambulance arrival to patient discharge), and case length (ie, time from call to patient discharge). Data were extracted directly from medical records using an automated reporting system. However, certain variables, such as “assessment” and “management” were manually coded by SV and CS and categorized to summarize the data to describe the paramedics’ provisional diagnosis. Excluded cases (eg, clearly irrelevant diagnoses) were identified using an automated reporting system and manual review.

### Statistical Methods

Continuous variables, except time-variables, were reported as mean (standard deviation, SD) and categorical variables were reported as frequency (%). Non-parametric variables (ie, time-variables [eg, duration of care]), were reported as median (interquartile range, IQR). All records obtained from January 2017 through December 2022 were used to describe call characteristics, provisional diagnoses, patient profile, and ambulance response times. Only records that received a diagnosis of “spinal pain” were used to describe care delivered by paramedics. The research team used multivariable logistic regression and developed three models to identify factors independently associated with each outcome: received ambulance transport to hospital (model one), received opioid medication (model two), and received opioid + anesthetic/benzodiazepine (model three). Patient characteristics such as age (>65 years), gender, socioeconomic status, baseline pain score, and time of call were force-entered as predictors to examine the association with each outcome. Additionally, opioid medication and combination medicines were included as predictors in model one (ambulance transport to hospital). The selection of potential predictors was theory-driven, based on the expertise of the research team and the available literature. For example, a previous study has shown that patients aged over 40 years of age and females are more likely to use ambulance service.^13^ It was hypothesized that patient characteristics such as older age and lower socioeconomic status would be associated with greater ambulance transport to hospital and opioid administration. Odds ratio (OR) and 95% confidence intervals (CIs) were reported. No statistical corrections were applied to control for family-wise error rate. The confidence intervals presented are uncorrected and should be interpreted for hypothesis generation rather than confirmatory inference. The Variance Inflation Factor (VIF) was used to measure multicollinearity in regression analysis. When VIF is greater than four, it is considered as the existence of multicollinearity. Cases that contained missing data were excluded from analysis. All analyses were conducted in Stata V17.0 (StataCorp; College Station, Texas USA).

## Results

### Triple-Zero Calls for Ambulance

**Figure 1.**
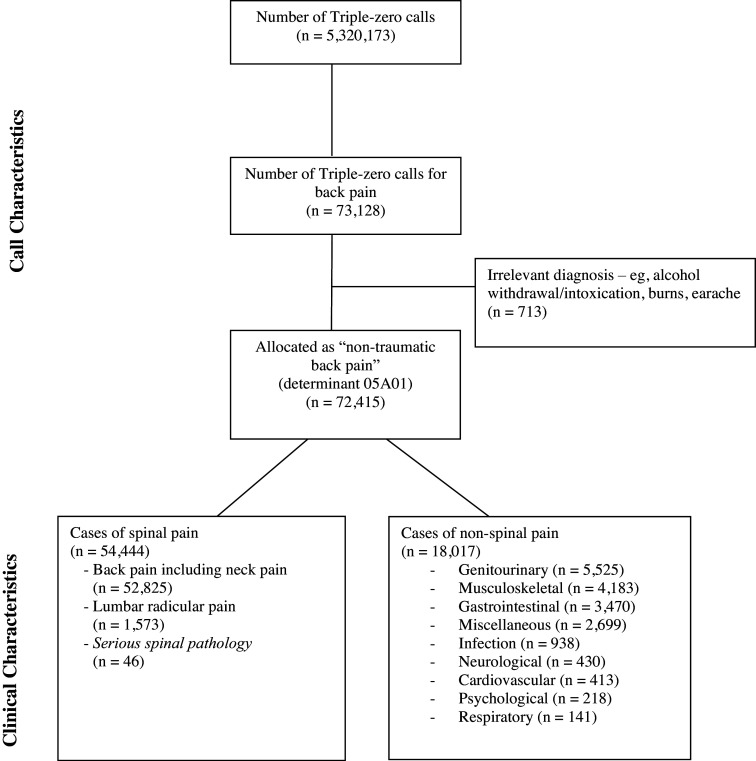
Call and Clinical Characteristics for Back Pain Complaints to Ambulance Service. Note: Cases of *serious spinal pathology* were not included in the analysis due to these cases likely representing different patient characteristics.

From January 1, 2017 through December 31, 2022, there were 5,320,173 calls to NSW Ambulance (Figure 1). Across the six-year period, there were 73,128 (1.4%) calls that had a chief complaint of back pain. The majority (68.4%) of “back-pain” incidents occurred “after-hours” and nearly all calls (98%) received an ambulance response within a 60-minute timeframe (Table 1). The median time between call to ambulance service and ambulance arrival on scene was 13.0 minutes (IQR 4.0 to 29.0 minutes). The median duration of care was 21.4 minutes (IQR 13.9 to 33.2 minutes) and the total time from call to ambulance service to patient discharge was 88.4 minutes (IQR 68.3 to 111.6 minutes). Following paramedic assessment and management, the disposition for the majority (80.7%) of patients was conveyance by ambulance to an emergency department for further assessment and management.

**Table 1.** Call Characteristics for Complaints Categorized by Ambulance Service as Spinal Pain

Call Characteristics	Spinal Pain	Non-Spinal Pain	Total
Volume of Calls n (%)	54,398 (74·4%)	18,730 (25·6%)	73,128 (100.0%)
**Time of Call**			
During Working Hours, n (%)	17,370 (75·3%)	5,706 (24·7%)	23,076 (31·6%)
After Hours (including weekend), n (%)	37,028 (74·0%)	13,024 (26·0%)	50,052 (68·4%)
**Response Time**			
Time from Call to Ambulance Dispatch, min; median (IQR)	9.0 (4.0 to 29.0)	9.0 (4.0 to 28.0)	9.0 (4.0 to 29.0)
Time from Ambulance Dispatch to Scene Arrival, min; median (IQR)	13.0 (8.0 to·21.0)	13.0 (8.0 to 20.0)	13.0 (8.0 to 21.0)
Ambulance Response Time (ie, time between call and ambulance arrival), min; median (IQR)	26.6 (15.7 to 45.4)	26.0 (15.6 to 44.4)	26.5 (15.7 to 45.1)

### Paramedic Assessment of Triple-Zero Calls for “Back Pain”

Of the 73,128 calls with a chief complaint of back pain, 713 were excluded due to having a diagnosis clearly irrelevant to back pain (eg, earache, alcohol withdrawal). From the 72,415 remaining cases, 54,444 (75.1%) were diagnosed by a paramedic as having spinal pain and 18,017 (24.9%) cases were due to non-spinal pain (Figure 1). Of those categorized as having spinal pain, 52,825 (97.0%) were due to back and/or neck pain, 1,573 (2.9%) were due to lumbar radicular pain, and 46 (0.1%) were due to serious spinal pathology. Genitourinary (eg, renal calculi), non-spinal musculoskeletal (eg, hip pain), gastrointestinal (eg, bowel obstruction), miscellaneous complaints (eg, headache), and infection made up 93% of non-spinal pain cases.

### Characteristics of Patients Who Called Ambulance Service with “Back Pain”

The mean age of people who called ambulance service with a chief complaint of back pain was 61.4 (SD = 19.9) years (Table 2). Women (53.7%) were slightly more likely to use ambulance services than men. Two-thirds of people who called the ambulance service with a chief complaint of back pain were from lower socioeconomic areas and 5.6% of cases were transferred for secondary-triage. Of cases where paramedics undertook a face-to-face assessment, 81.2% of patients were conveyed to an emergency department. People with a chief complaint of back pain were referred to extended-care paramedics (ie, specialist paramedics with additional clinical roles to manage lower-acuity conditions within the community) in 6.4% of cases, and 3.9% of cases were advised to seek alternative care (eg, general practice). The mean pain score of people who called the ambulance service with a chief complaint of back pain was 6.5 (SD = 2.9) out of ten. Mild pain (score of one-to-three out of ten) was reported by 18% of patients, moderate pain (score of four-to-seven out of ten) was reported by 34.1% of patients, and severe pain (a score of eight or higher out of ten) was reported by 47.9% of patients. People with spinal pain reported having a higher baseline pain score (6.7 out of ten; SD = 2.7) compared to those with non-spinal pain (5.7 out of ten; SD = 3.3). A higher proportion of people with spinal pain (50.3% of patients) reported severe pain compared to people with non-spinal pain (40.6% of patients).

**Table 2.** Characteristics of Patients Presenting to Paramedics with Spinal Pain

Characteristics	Total	Non-Spinal Pain	Spinal Pain		
			Back Pain (including neck pain)	Lumbar Radicular Pain	Total
**Total, n (%)**	73,128 (100.0%)	18,730 (25·6%)	52,825 (72·2%)	1,573 (2·2%)	54,398 (74·4%)
Age (years), mean (SD)	61·4 (SD = 19·9)	61·9 (SD = 20·2)	61·1 (SD = 19·8)	65·2 (SD = 18·5)	61·3 (SD = 19·7)
Female, n (%)	39,260 (53·7%)	9,932 (53·0%)	28,450 (53·9%)	878 (55·7%)	29,328 (53·9%)
Socioeconomic Status					
Low, n (%)	44,162 (60·7%)	11,462 (61·5%)	31,731 (60·4%)	969 (61·9%)	32,700 (60·4%)
High, n (%)	28,620 (39·3%)	7,182 (38·5%)	20,842 (39·6%)	596 (38·1%)	21,438 (39·6%)
Transferred to Secondary Triage, n (%)	4,112 (5·6%)	566 (3·0%)	3,468 (6·6%)	78 (5·0%)	3,546 (6·5%)
Received Ambulance Dispatch, n (%)	73,077 (100.0%)	18,717 (100.0%)	52,789 (100.0%)	1,571 (99.9%)	54,360 (100.0%)
Received Ambulance Transport, n (%)	59,377 (81·2%)	15,498 (82·8%)	42,575 (80·6%)	1,304 (83·0%)	43,879 (80·7%)
Referral					
To ECP, n (%)	4,677 (6·4%)	194 (1·0%)	4,434 (8·4%)	49 (3·1%)	4,483 (8·2%)
To Alternative Care, n (%)	2,855 (3·9%)	588 (3·1%)	2,189 (4·1%)	78 (5·0%)	2,267 (4·2%)
Pain Intensity					
Baseline Pain Intensity, (NPRS 0-10), mean (SD)	6·5 (SD = 2·9)	5·7 (SD = 3·3)	6·7 (SD = 2·7)	6·5 (SD = 3·0)	6·7 (SD = 2·7)
Mild (NPRS 0-3), n (%)	11,920 (18·0%)	4,720 (29·0%)	6,938 (14·3%)	262 (18·4%)	7,200 (14·4%)
Moderate (NPRS 4-7), n (%)	22,674 (34·1%)	4,971 (30·5%)	17,213 (35·4%)	490 (34·4%)	17,703 (35·3%)
Severe (NPRS 8-10), n (%)	31,816 (47·9%)	6,615 (40·6%)	24,528 (50·4%)	673 (47·2%)	25,201 (50·3%)
Priority Code					
P1: Immediate Response (<30 minutes), n (%)	1,372 (1·9%)	372 (2·0%)	967 (1·8%)	33 (2·1%)	1,000 (1·8%)
P2: Response within 60 Minutes, n (%)	71,717 (98·1%)	18,349 (98·0%)	51,829 (98·2%)	1,539 (97·9%)	53,368 (98·2%
Duration of Care, min median (IQR)	21.4 (13.9 to 33.2)	19.0 (12.0 to 29.4)	22.2 (14.5 to 34.8)	21.3 (14.4 to 32.8)	22.2 (14.5 to 34.7)
Case Length, min median (IQR)	88.4 (68.3 to 111.6)	84.8 (65.3 to 107.4)	89.6 (69.2 to 113.2)	88.8 (67.8 to 111.2)	89.6 (69.2 to 113.2)

Abbreviations: EPC, extended-care paramedic; NPRS, numeric pain rating scale.

### Non-Pharmacological Care

Advice and reassurance were documented to 65.4% of patients (Table 3). Patient positioning (ie, helping a patient into a more comfortable position to relieve their pain) was documented in 4.4% of patients with spinal pain. Education, exercise, and heat were recorded to be provided to 0.1%, 0.4%, and 0.2% of patients, respectively.

**Table 3.** Management of Spinal Pain by Ambulance Services

Care Delivery	Overall, n (%)	Back Pain (including neck pain), n (%)	Lumbar Radicular Pain, n (%)
**Total**	**54,084 (100%)**^a^	**52,519 (97**·**1%)**	**1,565 (2·9%)**
**Non-Pharmacological Care**			
Advice and Reassurance, n (%)	35,342 (65·4%)	34,238 (65·2%)	1,104 (70·5%)
Education, n (%)	29 (0·1%)	28 (0·1%)	1 (0·1%)
Exercises, n (%)	233 (0·4%)	231 (0·4%)	2 (0·1%)
Patient Positioning, n (%)	2,380 (4·4%)	2,298 (4·4%)	82 (5·2%)
Heat Therapy, n (%)	108 (0·2%)	107 (0·2%)	1 (0·1%)
Cold Therapy, n (%)	64 (0·1%)	64 (0·1%)	0 (0·0%)
**Pharmacological Care**			
Paracetamol, n (%)	8,789 (16·3%)	8,613 (16·4%)	176 (11·3%)
NSAIDs, n (%)	7,988 (14·8%)	7,830 (14·9%)	158 (10·1%)
Methoxyflurane, n (%)	18,025 (33·3%)	17,551 (33·4%)	474 (30·3%)
Anesthetics, n (%)	159 (0·3%)	153 (0·3%)	6 (0·4%)
Opioids, n (%)	20,236 (37·4%)	19,727 (37·6%)	509 (32·5%)
Morphine	14,505 (26·8%)	14,106 (26·9%)	399 (25·5%)
Oxycodone	3,119 (5·8%)	3,092 (5·9%)	27 (1·7%)
Fentanyl	2,735 (5·1%)	2,646 (5·0%)	89 (5·7%)
Paracetamol-Codeine	383 (0·7%)	379 (0·7%)	4 (0·2%)
Benzodiazepine, n (%)	270 (0·5%)	263 (0·5%)	7 (0·5%)
**Combined Medicines**			
Paracetamol and Ibuprofen, n (%)	11,442 (21·2%)	11,209 (21·3%)	233 (14·9%)
Paracetamol or Ibuprofen and Methoxyflurane, n (%)	2,494 (4·6%)	2,453 (4·7%)	41 (2·6%)
Paracetamol or Ibuprofen and Opioid, n (%)	3,653 (6·8%)	3,600 (6·9%)	53 (3·4%)
Methoxyflurane and Opioid, n (%)	5,768 (10·7%)	5,615 (10·7%)	153 (9·8%)
Opioid and Benzodiazepine, n (%)	249 (0·5%)	242 (0·5%)	7 (0·5%)

Abbreviation: NSAID, non-steroidal anti-inflammatory drugs.

a There were 314 cases with missing data leaving 54,084 patients with spinal pain for analysis.

### Pharmacological Care

Opioids (37.4% of patients) and methoxyflurane (33.3% of patients) were the most administered medicines for people with spinal pain. Of the opioids, morphine was the most used (26.8% of patients) and very few people with spinal pain received benzodiazepines (0.5% of cases) or other anesthetics (0.3%). Approximately 20% of patients with spinal pain received a combination of paracetamol and NSAIDs and 10.7% of patients received methoxyflurane and opioids.

Paracetamol was the most frequent medication administered for mild spinal pain, followed by NSAIDs (16.2% and 13.9% of patients with mild pain, respectively). Of the patients with moderate spinal pain, 27.0% received methoxyflurane, and 24.4% were administered opioids. Opioids were administered to 57.9% of patients with severe spinal pain and methoxyflurane to 47.4% of patients (Supplementary File 1; available online only).

### Multivariable Regression

Older age (OR = 1.36), moderate to severe spinal pain intensity (OR = 1.52–3.40), presentations outside of working hours (OR = 1.1), and opioid administration (OR = 1.29) increased the odds of ambulance transport to the emergency department, whereas patients from higher socioeconomic areas were less likely to be transported (OR = 0.86; Table 4). Factors associated with opioid administration included male sex (OR = 1.26), higher socioeconomic status (OR = 1.05), and moderate to severe spinal pain intensity (OR = 4.39–18.90). However, patients presenting “after-hours” were 0.85-times less likely to be administered opioids. Similar factors were associated with administration of combination medicines apart from age where older patients were less likely to be administered opioids plus anesthetics or benzodiazepines (OR = 0.53).

**Table 4.** Association between Patient Characteristics and Type of Care for Spinal Pain

Predictor Factors	Received Ambulance Transport to Hospital (Model 1)		Received an Opioid (Model 2)		Received Combination Medicines (Opioid + Anesthetic OR Opioid + Benzodiazepine (Model 3)	
	OR	Unadjusted 95% CI	OR	Unadjusted 95% CI	OR	Unadjusted 95% CI
Age (≥ 65 years)	1·36	1·30 to 1·44	0·99	0·95 to 1·03	0·53	0·50 to 0·58
Male	1·03	0·98 to 1·08	1·26	1·21 to 1·31	1·26	1·17 to 1·35
Socioeconomic Status (higher socioeconomic status)	0·86	0·82 to 0·90	1·05	1·01 to 1·10	1·44	1·35 to 1·54
Moderate Pain	1·52	1·43 to 1·63	4·39	4·00 to 4·84	2·94	2·44 to 3·56
Severe Pain	3·40	3·16 to 3·65	18·90	17·18 to 20·79	5·89	4·91 to 7·07
Presentation After Working Hours	1·10	1·05 to 1·16	0·85	0·81 to 0·89	0·67	0·62 to 0·71
Received Opioid	1·29	1·21 to 1·38				
Received Opioid + Anesthetic or Benzodiazepine	0·12	0·11 to 0·13				

## Discussion

### Primary Findings

In NSW, non-traumatic back pain complaints contributed to 1.4% of the total ambulance service caseload with 60.1% of presentations coming from lower socioeconomic areas. Three-quarters of people seeking care with a chief complaint of back pain who were triaged via the call-taker as having non-traumatic back pain were diagnosed by paramedics as having pain of spinal origin. Oftentimes, calls occurred “after-hours,” and where paramedics were dispatched, they arrived on scene approximately 15 minutes after the initial call. Spinal pain encountered by paramedics was almost entirely simple back and neck pain, with seldom lumbar radicular pain, and very rarely serious spinal pathology. Paramedic care of spinal pain typically includes advice and reassurance, and pain medicine administration, of which opioids were the most administered medication. Very rarely does care include education, exercises, or heat therapy. Older age, moderate-severe spinal pain, opioid administration, and presentation outside of working hours were associated with ambulance transport to the emergency department.

This is the first study in Australia to describe the management of spinal pain by paramedics and includes all calls to ambulance service with a chief complaint of back pain. The diagnoses made by paramedics were used to determine the proportion of patients presenting to ambulance service with spinal and non-spinal pain, and to describe care delivered specifically to patients with back and/or neck pain and lumbar radicular pain. A previous study in England evaluated calls to ambulance services for lower back pain across a 12-month period, but health service delivery was not categorically explored for the different types of spinal conditions.^13^ This study reports approximately 40% more back pain cases are of spinal origin, and 12% more spinal pain cases are transported to hospital. Opioid medicines and inhaled analgesics (ie, methoxyflurane) were administered to people with spinal pain more in Australia than those in the English study.^13^ For example, the current study suggests that 37% of patients received opioid medicines compared to only 13% in the English study.^13^ The variation between ambulance services management of spinal pain may be due to differences in ambulance service guideline recommendations (across jurisdictions) and access to alternate health services.

Back pain guidelines exist in NSW, but only for specialist paramedics (ie, extended-care paramedics) who are trained to manage patients with back pain in their home or within community health services, such as medical and allied health services, to reduce unnecessary ambulance transport to hospitals. The findings in this study suggest that only 8.2% of patients with spinal pain are being managed by specialist paramedics who use back-pain-specific guidelines, which may highlight a shortage in the extended care workforce, or a gap in practice between getting the most appropriate paramedic to the right presentation (eg, back pain) in which they receive additional training. The other 90% of patients with spinal pain are being managed by non-specialist paramedics through generic pain management guidelines, which might explain why one-third of patients with back pain in Australian emergency departments arrived via ambulance transport.^5,6^

Australian ambulance service guidelines recommend a stepwise approach to pain management.^18^ Step one involves non-pharmacological care such as advice/reassurance and heat/cold therapy, and steps two and three recommend the use of medication to manage a person’s pain. For mild to moderate pain (step two), paracetamol, NSAIDs, and methoxyflurane are recommended, and opioids are recommended for severe pain (step three).^18^ This study showed that paramedic care delivered to patients with spinal pain is mostly in accordance with current ambulance service pain management guidelines. For example, only 31% of patients with mild or moderate spinal pain received an opioid, whereas around 58% of patients with severe pain were administered an opioid medicine. However, the findings also highlight the limited scope of medications (eg, paracetamol, ibuprofen, opioids, and methoxyflurane) that paramedics can access to manage a person’s pain, which may explain the high number of patients receiving opioid medicines.

One discrepancy between the findings in this study, current ambulance service guidelines, and primary care guidelines for the management of back pain is the use of heat therapy.^12^ The findings in this study show that less than one percent of patients with spinal pain had superficial heat documented on their medical records, despite NSW Ambulance service guidelines recommending heat therapy. Evidence from a small randomized trial (n = 90) has shown that patients with acute low back pain who received heat (ie, active warming via an electric blanket) during ambulance transport had a greater reduction in pain intensity using a 100-point visual analogue pain scale compared to patients who received sham treatment (mean difference 32.2; 95% CI, 25.7 to 38.7).^19^ Policymakers should revise the reporting of routinely-collected data and make all variables relating to care delivered, including non-pharmacological treatments, to be documented. Additionally, future research should evaluate the feasibility and effectiveness of superficial heat (ie, heat wrap therapy) in ambulance service for the management of non-traumatic back pain and explore reasons for ambulance service use in lower socioeconomic areas.

## Limitations

A limitation of this study is the potential misclassification of triple-zero calls for back pain and the type of spinal pain by paramedics. The paramedic’s diagnosis was used in this study, which is based upon an “out-of-hospital” history and physical assessment but is not informed by subsequent testing and diagnosis in the emergency department (eg, diagnostic imaging or laboratory tests). This might explain the lower rate of serious spinal pathology (0.1%), which is lower than what is typically observed in primary care (∼1.0%)^20^ or an emergency department setting (2.0% to 5.0%) in patients with back pain of spinal origin.^6,21^ A second limitation is that there is variation in medical record coding and not all variables within the ambulance electronic medical record dataset require mandatory reporting. For example, pharmacological aspects of care (eg, dosage, route of administration) are mandatory, though variables relating to non-pharmacological care are not. Therefore, paramedics may have provided non-pharmacological care to a patient without documenting it in the medical record. This may explain the apparent low rates of use of non-pharmacological care options (eg, education, heat/cold therapy). Moreover, as with retrospective study designs, there may be other potential confounders amongst patients that the research team were not aware of and thus would not have been controlled for in this model.

## Conclusion

Non-traumatic back pain is a common presentation to ambulance services with patients accessing ambulance services predominantly outside of working hours. Three-quarters of calls for non-traumatic back pain were thought to be of spinal origin following paramedic assessment. Opioids and methoxyflurane were administered to at least one-third of patients. The final disposition for eight out of ten patients was conveyance to an emergency department.

## Supporting information

Vella et al. supplementary material Vella et al. supplementary material

## Supplementary Materials

To view supplementary material for this article, please visit https://doi.org/10.1017/S1049023X25000251
