# Supplementary material for: Paramedic Management of Non-Traumatic Back Pain in a Large Australian Ambulance Service: A Retrospective Study
Source: Prehosp Disaster Med. 2025 Apr 7;40(2):77–85. doi: 10.1017/S1049023X25000251 (PMC12018008; doi:10.1017/S1049023X25000251)
Supplement: Vella et al. supplementary material [file S1049023X25000251sup001.docx]

| **Baseline pain score (0-10)** | **Mild pain (1-3), n (%)** | **Moderate pain (4-7), n (%)** | **Severe pain (8-10), n (%)** |
| --- | --- | --- | --- |
| Overall, n = 49,925 | 7,157 (14.3%) | 17,603 (35.3%) | 25,165 (50.4%) |
| Paracetamol | 1,162 (16.2%) | 3,435 (19.5% | 3,457 (13.7%) |
| NSAIDs | 993 (13.9%%) | 3,155 (17.9%) | 3,211 (12.8%) |
| Methoxyflurane | 638 (8.9%) | 4,750 (27.0%) | 11,930 (47.4%) |
| Anaesthetics | 2 (0.0%) | 10 (0.1%) | 139 (0.6%) |
| Opioids | 497 (6.9%) | 4,297 (24.4%) | 14,569 (57.9%) |
| Benzodiazepines | 10 (0.1%) | 37 (0.2%) | 210 (0.8%) |

**Supplementary File 1. Medications administered to patients with spinal pain (including neck pain) according to their pain intensity level**

Abbreviation: NSAID; non-steroidal anti-inflammatory drug.

* patients could have been administered multiple medications.
